# Supplementary material for: A Likelihood Approach for Real-Time Calibration of Stochastic Compartmental Epidemic Models
Source: PLoS Comput Biol. 2017 Jan 17;13(1):e1005257. doi: 10.1371/journal.pcbi.1005257 (PMC5240920; doi:10.1371/journal.pcbi.1005257)
Supplement: S1 File — (TAR.GZ) [file pcbi.1005257.s014.tar.gz › HSPH_Online-SI-Revision/Additions/EnKF08/n100-extreme_noisy/n100-extreme_noisy_EnKF2_table-4.pdf]

1	newly 3 weeks spec	10 775.3	939.96	{29., 4335.}	91.2767	1.70448	newly 3 weeks cumul	25 108.6	6409.57	{309., 12 293.}	88.51	1.13244	newly 5 weeks spec	10 934.9	342.72	{0., 898.}	96.8658	2.49214	newly 5 weeks cumul	47 514.6	7310.97	{381., 19 249.}	87.1678	1.31036	newly 58 weeks cumul	88 201.8	7812.97	{427., 24 739.}	91.3711	1.52481
2	newly 3 weeks spec	8383.	605.44	{13., 829.}	92.7778	1.5878	newly 3 weeks cumul	21 608.	16 924.7	{1108., 19 742.}	117.618	0.719785	newly 5 weeks spec	8027.	93.43	{0., 238.}	98.8361	2.9004	newly 5 weeks cumul	38 163.	17 236.9	{1108., 20 536.}	100.839	0.896695	newly 54 weeks cumul	72 415.	17 329.8	{1108., 20 635.}	92.0659	1.11871
3	newly 3 weeks spec	12 915.	1837.68	{32., 6309.}	88.385	1.61628	newly 3 weeks cumul	29 820.	8918.17	{155., 20 375.}	92.7746	1.18547	newly 5 weeks spec	11 421.	608.21	{4., 1852.}	94.8412	2.20141	newly 5 weeks cumul	54 424.	10 568.2	{180., 29 662.}	88.4508	1.34944	newly 59 weeks cumul	84 212.	11 614.9	{206., 36 202.}	87.3208	1.48203
4	newly 3 weeks spec	15 590.	1785.	{0., 6567.}	88.5507	2.67894	newly 3 weeks cumul	27 674.	33 124.7	{6623., 71 739.}	65.5031	0.294684	newly 5 weeks spec	15 345.	516.88	{0., 2066.}	96.6316	3.42713	newly 5 weeks cumul	61 225.	34 525.6	{6908., 71 739.}	57.6643	0.446214	newly 63 weeks cumul	88 766.	35 810.	{6958., 71 739.}	63.1261	0.550866
5	newly 3 weeks spec	10 521.	1942.49	{0., 4560.}	94.8669	2.25096	newly 3 weeks cumul	21 016.	6490.2	{1086., 17 739.}	87.8199	0.982404	newly 5 weeks spec	12 782.	1159.52	{0., 1348.}	91.8733	3.02672	newly 5 weeks cumul	46 785.	9306.79	{1086., 29 020.}	87.0195	1.24327	newly 60 weeks cumul	85 329.	11 282.8	{1086., 55 655.}	86.7773	1.45248
6	newly 3 weeks spec	10 702.	916.04	{20., 2253.}	92.4177	1.80011	newly 3 weeks cumul	23 963.	5212.81	{135., 10 255.}	89.1209	1.31326	newly 5 weeks spec	11 271.	299.25	{1., 991.}	97.345	2.46973	newly 5 weeks cumul	47 247.	6030.03	{165., 12 748.}	89.3247	1.51154	newly 58 weeks cumul	86 171.	6544.7	{180., 14 958.}	92.8314	1.72198
7	newly 3 weeks spec	11 555.	2757.04	{66., 15 560.}	86.6164	1.37978	newly 3 weeks cumul	26 529.	16 526.5	{653., 63 779.}	99.4889	0.865666	newly 5 weeks spec	11 237.	870.42	{1., 3161.}	92.254	2.18086	newly 5 weeks cumul	49 918.	18 924.5	{727., 79 415.}	88.3411	1.0269	newly 56 weeks cumul	84 780.	20 586.2	{729., 80 432.}	82.2699	1.17122
8	newly 3 weeks spec	11 018.	815.09	{18., 815.}	93.347	1.63764	newly 3 weeks cumul	24 447.	5125.46	{512., 5056.}	85.4825	1.0174	newly 5 weeks spec	11 207.	312.55	{0., 513.}	97.2111	2.43446	newly 5 weeks cumul	47 781.	5927.6	{571., 5149.}	88.378	1.2273	newly 58 weeks cumul	87 124.	6781.87	{728., 6024.}	92.2158	1.43374
9	newly 3 weeks spec	10 870.	1440.9	{32., 924.}	91.8033	1.42684	newly 3 weeks cumul	22 125.	7472.96	{2319., 16 791.}	85.6253	0.807489	newly 5 weeks spec	12 694.	404.21	{0., 597.}	96.8157	2.28843	newly 5 weeks cumul	47 679.	8657.24	{2822., 16 810.}	85.6209	1.03756	newly 58 weeks cumul	88 989.	9268.63	{2838., 16 810.}	90.0016	1.25614
10	newly 3 weeks spec	15 442.	935.42	{215., 1651.}	93.9424	1.41718	newly 3 weeks cumul	29 673.	20 501.9	{8784., 48 647.}	66.7771	0.422855	newly 5 weeks spec	11 765.	142.04	{3., 201.}	98.7927	2.62676	newly 5 weeks cumul	57 244.	20 966.7	{8926., 48 694.}	67.3908	0.602709	newly 62 weeks cumul	72 720.	21 187.5	{8938., 48 695.}	71.4303	0.688152
11	newly 3 weeks spec	9197.	2551.26	{57., 6129.}	91.2115	1.26969	newly 3 weeks cumul	23 145.	16 368.3	{325., 85 097.}	101.473	0.806858	newly 5 weeks spec	8893.	824.5	{1., 1411.}	90.8793	1.98847	newly 5 weeks cumul	41 559.	18 594.4	{387., 95 546.}	92.4062	0.959313	newly 56 weeks cumul	75 767.	20 986.5	{410., 95 751.}	83.9442	1.11542
12	newly 3 weeks spec	11 654.	2760.67	{91., 13 343.}	87.3062	1.3228	newly 3 weeks cumul	24 296.	13 107.	{355., 29 142.}	106.501	1.09548	newly 5 weeks spec	12 745.	1201.63	{44., 2463.}	91.217	1.64799	newly 5 weeks cumul	50 388.	16 047.1	{468., 57 435.}	91.5194	1.2244	newly 57 weeks cumul	88 506.	23 891.4	{637., 88 455.}	78.5906	1.16307
13	newly 3 weeks spec	8371.	1199.09	{0., 1211.}	91.636	2.65757	newly 3 weeks cumul	19 985.	14 399.2	{1210., 14 844.}	90.7906	0.607292	newly 5 weeks spec	8948.	272.37	{0., 537.}	96.9561	3.05231	newly 5 weeks cumul	38 014.	15 171.3	{1610., 18 781.}	86.2268	0.789816	newly 55 weeks cumul	83 218.	16 104.1	{2391., 18 781.}	83.7752	1.01865
14	newly 3 weeks spec	11 434.	1042.59	{0., 952.}	95.9198	2.47998	newly 3 weeks cumul	18 644.	5957.29	{480., 18 296.}	93.0711	1.09385	newly 5 weeks spec	15 310.	507.9	{0., 309.}	96.6826	3.27641	newly 5 weeks cumul	50 732.	7225.49	{598., 25 938.}	89.3932	1.43422	newly 60 weeks cumul	70 240.	8236.35	{687., 25 938.}	89.0858	1.54535
15	newly 3 weeks spec	13 412.	1498.94	{1., 861.}	96.4872	2.05835	newly 3 weeks cumul	27 516.	7478.3	{731., 18 215.}	92.1327	1.06712	newly 5 weeks spec	12 056.	465.91	{0., 280.}	96.1355	2.76103	newly 5 weeks cumul	54 113.	8871.99	{776., 18 215.}	91.6652	1.29459	newly 60 weeks cumul	75 951.	9377.69	{781., 18 215.}	90.6744	1.40571
16	newly 3 weeks spec	15 726.	812.2	{0., 618.}	96.1872	2.50598	newly 3 weeks cumul	30 526.	5012.98	{868., 7070.}	89.6953	1.19643	newly 5 weeks spec	14 011.	345.81	{0., 237.}	97.7383	3.10199	newly 5 weeks cumul	61 514.	5906.9	{877., 7555.}	92.3921	1.45359	newly 60 weeks cumul	88 752.	6426.44	{877., 8345.}	93.2036	1.59088
17	newly 3 weeks spec	9053.	640.85	{0., 1272.}	92.9211	2.79355	newly 3 weeks cumul	21 971.	22 209.8	{1164., 98 513.}	101.618	0.511479	newly 5 weeks spec	9419.	115.37	{0., 250.}	98.7751	3.31865	newly 5 weeks cumul	40 887.	22 557.2	{1639., 98 513.}	88.3787	0.67915	newly 55 weeks cumul	79 924.	22 897.5	{2728., 98 513.}	81.9115	0.860909
18	newly 3 weeks spec	8959.	1906.56	{25., 1547.}	90.4181	1.37219	newly 3 weeks cumul	19 558.	14 016.1	{226., 16 875.}	96.9941	0.714938	newly 5 weeks spec	10 392.	614.23	{1., 905.}	94.0894	2.38778	newly 5 weeks cumul	40 376.	15 668.5	{231., 22 945.}	90.7801	0.944872	newly 56 weeks cumul	75 084.	16 770.9	{231., 22 978.}	87.4274	1.14943
19	newly 3 weeks spec	11 412.	1135.61	{19., 5524.}	90.049	1.56681	newly 3 weeks cumul	23 492.	7650.34	{117., 41 027.}	85.4631	0.957796	newly 5 weeks spec	12 862.	359.63	{0., 940.}	97.2039	2.42935	newly 5 weeks cumul	49 674.	8634.26	{148., 43 664.}	83.0209	1.16441	newly 58 weeks cumul	89 565.	9385.32	{185., 44 010.}	89.5212	1.36697
20	newly 3 weeks spec	15 824.	2762.24	{3., 8411.}	83.6299	1.96025	newly 3 weeks cumul	21 990.	35 989.5	{6644., 82 169.}	89.5035	0.280388	newly 5 weeks spec	20 039.	1143.21	{0., 3175.}	94.2951	3.24318	newly 5 weeks cumul	66 304.	38 838.5	{6925., 82 426.}	58.2575	0.421739	newly 63 weeks cumul	91 232.	42 039.6	{6956., 82 426.}	60.2546	0.487244
21	newly 3 weeks spec	11 565.	1551.66	{0., 1985.}	95.6642	2.23526	newly 3 weeks cumul	23 774.	7200.83	{1059., 23 817.}	92.9241	1.08985	newly 5 weeks spec	12 361.	656.47	{0., 224.}	95.2115	3.0572	newly 5 weeks cumul	49 979.	8939.75	{1066., 25 295.}	90.2903	1.33624	newly 60 weeks cumul	80 337.	10 111.	{1066., 26 545.}	88.0885	1.49046
22	newly 3 weeks spec	13 043.	226.68	{0., 495.}	98.2621	2.57192	newly 3 weeks cumul	25 673.	7976.2	{737., 19 659.}	75.5634	0.756511	newly 5 weeks spec	13 126.	31.74	{0., 77.}	99.7582	3.57005	newly 5 weeks cumul	53 143.	8083.78	{762., 19 671.}	85.9265	1.0468	newly 61 weeks cumul	81 637.	8120.21	{764., 19 671.}	90.0533	1.22482
23	newly 3 weeks spec	8971.	703.84	{1., 946.}	95.6027	1.89671	newly 3 weeks cumul	22 691.	8402.34	{1265., 6879.}	91.1475	0.762903	newly 5 weeks spec	8717.	238.97	{0., 244.}	98.1024	2.77403	newly 5 weeks cumul	40 575.	9079.81	{1266., 7619.}	91.4882	0.965546	newly 54 weeks cumul	74 886.	9327.83	{1266., 7929.}	91.7108	1.19961
24	newly 3 weeks spec	9753.	804.5	{16., 811.}	96.0949	1.65545	newly 3 weeks cumul	22 644.	9422.15	{2862., 9446.}	93.03	0.767969	newly 5 weeks spec	10 377.	202.42	{0., 222.}	98.0493	2.80606	newly 5 weeks cumul	43 672.	10 025.1	{2862., 9550.}	90.7939	0.99897	newly 54 weeks cumul	86 096.	10 219.4	{2862., 9555.}	90.211	1.2565
25	newly 3 weeks spec	14 475.	2142.65	{112., 3275.}	90.2571	1.42628	newly 3 weeks cumul	28 620.	9350.32	{882., 7031.}	97.6974	1.092	newly 5 weeks spec	13 481.	881.21	{17., 2132.}	93.4633	1.76464	newly 5 weeks cumul	57 956.	11 571.	{1157., 17 444.}	91.1605	1.25252	newly 57 weeks cumul	86 463.	16 322.2	{1220., 85 026.}	84.4426	1.26827
26	newly 3 weeks spec	8715.	985.64	{0., 811.}	99.7356	3.0724	newly 3 weeks cumul	21 621.	12 582.7	{1921., 16 560.}	86.7967	0.630527	newly 5 weeks spec	8698.	221.69	{0., 540.}	97.4513	3.50734	newly 5 weeks cumul	39 423.	13 249.6	{2048., 16 608.}	84.5832	0.807184	newly 55 weeks cumul	81 661.	14 077.9	{2085., 34 379.}	83.9608	1.02774
27	newly 3 weeks spec	9364.	2458.36	{49., 15 179.}	91.9695	1.39351	newly 3 weeks cumul	24 919.	14 644.2	{1324., 83 406.}	105.444	0.868227	newly 5 weeks spec	8776.	819.98	{1., 1917.}	90.6595	2.28728	newly 5 weeks cumul	43 318.	16 856.1	{1343., 99 485.}	95.1103	1.01839	newly 56 weeks cumul	79 621.	18 240.7	{1343., 108 498.}	86.1412	1.19351
28	newly 3 weeks spec	11 755.	1879.88	{0., 2182.}	96.4721	2.17747	newly 3 weeks cumul	27 332.	6151.8	{626., 14 850.}	90.5343	1.15309	newly 5 weeks spec	11 093.	842.42	{0., 655.}	93.065	2.84606	newly 5 weeks cumul	50 746.	8410.74	{712., 17 934.}	89.5641	1.35163	newly 60 weeks cumul	87 654.	9722.36	{737., 18 597.}	88.9083	1.54375
29	newly 3 weeks spec	8104.	621.45	{0., 672.}	96.6605	2.24058	newly 3 weeks cumul	20 099.	12 232.2	{1239., 8730.}	111.83	0.818847	newly 5 weeks spec	8778.	191.4	{0., 222.}	98.5689	3.16269	newly 5 weeks cumul	37 699.	12 783.5	{1247., 8796.}	98.7109	1.02717	newly 54 weeks cumul	75 236.	12 975.1	{1247., 8799.}	91.4262	1.27307
30	newly 3 weeks spec	13 462.	2974.42	{1., 7737.}	80.0593	1.72564	newly 3 weeks cumul	25 138.	31 155.1	{6667., 66 931.}	68.3983	0.28994	newly 5 weeks spec	14 527.	1176.88	{0., 3553.}	91.8987	2.73651	newly 5 weeks cumul	55 394.	34 097.	{6885., 66 931.}	58.2996	0.418666	newly 63 weeks cumul	91 285.	37 225.3	{6916., 79 610.}	63.7317	0.550954
31	newly 3 weeks spec	13 467.	7124.7	{964., 20 560.}	66.8753	0.590505	newly 3 weeks cumul	21 982.	49 258.	{11 927., 108 008.}	152.823	0.411682	newly 5 weeks spec	17 282.	1676.05	{54., 3526.}	90.3018	1.4766	newly 5 weeks cumul	58 272.	54 266.9	{13 616., 122 994.}	64.4349	0.394921	newly 64 weeks cumul	87 865.	56 800.6	{13 776., 123 339.}	52.5061	0.428612
32	newly 3 weeks spec	11 691.	300.69	{0., 470.}	97.428	2.46152	newly 3 weeks cumul	23 586.	5877.24	{1283., 13 360.}	76.6571	0.799445	newly 5 weeks spec	12 032.	61.76	{0., 88.}	99.4867	3.28077	newly 5 weeks cumul	48 919.	6066.58	{1332., 14 367.}	87.5987	1.0932	newly 61 weeks cumul	74 173.	6145.57	{1337., 14 524.}	91.7145	1.2681
33	newly 3 weeks spec	9366.	1858.63	{111., 5823.}	90.4197	1.47004	newly 3 weeks cumul	20 595.	9423.23	{1283., 18 001.}	99.1521	0.998609	newly 5 weeks spec	10 722.	566.56	{3., 1611.}	94.7159	2.15022	newly 5 weeks cumul	42 102.	10 964.2	{1607., 24 671.}	91.0118	1.2011	newly 59 weeks cumul	87 634.	12 343.6	{1768., 48 999.}	87.796	1.43945
34	newly 3 weeks spec	6522.	1050.71	{34., 943.}	97.1201	1.37129	newly 3 weeks cumul	16 514.	8047.49	{2207., 9035.}	90.7167	0.675484	newly 5 weeks spec	7135.	332.62	{0., 187.}	97.9554	2.4369	newly 5 weeks cumul	30 872.	9021.2	{2318., 9777.}	91.0985	0.89468	newly 54 weeks cumul	67 088.	9326.08	{2343., 9860.}	90.7174	1.19182
35	newly 3 weeks spec	10 120.	1131.93	{76., 987.}	94.8705	1.54978	newly 3 weeks cumul	20 852.	4650.71	{1400., 3654.}	92.562	1.08112	newly 5 weeks spec	12 158.	397.53	{7., 542.}	96.7303	2.19621	newly 5 weeks cumul	45 344.	5750.52	{1504., 5418.}	92.3495	1.32576	newly 59 weeks cumul	90 553.	6584.59	{1505., 7501.}	92.7285	1.55636
36	newly 3 weeks spec	8518.	1868.34	{237., 1127.}	92.1672	1.1924	newly 3 weeks cumul	20 967.	9424.27	{1279., 12 887.}	104.808	0.96579	newly 5 weeks spec	8858.	653.48	{40., 539.}	94.0741	1.54915	newly 5 weeks cumul	39 044.	11 124.7	{1746., 20 258.}	95.358	1.0944	newly 57 weeks cumul	75 101.	12 779.1	{2276., 20 284.}	89.6792	1.21981
37	newly 3 weeks spec	14 436.	1033.47	{228., 1834.}	92.841	1.34946	newly 3 weeks cumul	27 190.	19 540.5	{6384., 44 414.}	67.9746	0.394681	newly 5 weeks spec	14 344.	208.9	{4., 242.}	98.5436	2.60252	newly 5 weeks cumul	57 920.	20 156.9	{8426., 44 456.}	69.8469	0.611922	newly 62 weeks cumul	85 971.	20 498.9	{8436., 44 456.}	76.2579	0.758196
38	newly 3 weeks spec	14 671.	1313.45	{296., 1946.}	91.0473	1.3237	newly 3 weeks cumul	23 153.	22 136.5	{8733., 65 959.}	74.696	0.370078	newly 5 weeks spec	16 782.	221.35	{5., 250.}	98.681	2.65496	newly 5 weeks cumul	59 731.	22 872.8	{8774., 66 071.}	65.8228	0.599907	newly 62 weeks cumul	80 717.	23 064.3	{8808., 66 071.}	71.4258	0.711123
39	newly 3 weeks spec	8107.	2254.44	{26., 5199.}	89.8976	1.26027	newly 3 weeks cumul	18 981.	14 211.8	{245., 42 467.}	96.2017	0.725131	newly 5 weeks spec	8590.	816.12	{1., 1004.}	91.2102	2.12335	newly 5 weeks cumul	36 334.	16 368.9	{260., 50 782.}	88.8053	0.909882	newly 56 weeks cumul	64 192.	18 043.5	{262., 67 420.}	84.319	1.08195
40	newly 3 weeks spec	9375.	1933.54	{228., 3412.}	89.8057	1.18809	newly 3 weeks cumul	23 924.	11 941.4	{1669., 12 082.}	110.483	0.95168	newly 5 weeks spec	8935.	686.1	{35., 1370.}	94.4584	1.57027	newly 5 weeks cumul	42 535.	13 671.9	{2134., 16 250.}	97.8476	1.07015	newly 57 weeks cumul	74 746.	16 192.1	{2548., 85 583.}	89.4932	1.18233
41	newly 3 weeks spec	9002.	1142.75	{18., 4691.}	87.6651	1.63158	newly 3 weeks cumul	21 615.	7574.32	{150., 35 892.}	91.5052	1.2034	newly 5 weeks spec	8585.	511.8	{0., 1291.}	94.0384	2.27621	newly 5 weeks cumul	39 635.	8795.68	{185., 38 628.}	84.2541	1.34178	newly 58 weeks cumul	64 657.	10 575.7	{231., 42 656.}	84.8383	1.48105
42	newly 3 weeks spec	13 145.	1350.96	{273., 2178.}	89.7226	1.21034	newly 3 weeks cumul	25 907.	24 496.3	{8367., 62 975.}	76.5107	0.383589	newly 5 weeks spec	11 817.	198.79	{5., 274.}	98.3178	2.4058	newly 5 weeks cumul	52 038.	25 181.4	{8659., 65 152.}	65.1899	0.53316	newly 62 weeks cumul	70 332.	25 347.1	{8670., 65 325.}	68.4528	0.632469
43	newly 3 weeks spec	10 255.	1402.94	{0., 1433.}	96.1708	2.88428	newly 3 weeks cumul	22 579.	11 760.9	{1264., 14 653.}	74.9094	0.582677	newly 5 weeks spec	11 209.	450.39	{0., 849.}	95.9819	3.21083	newly 5 weeks cumul	45 260.	12 964.6	{1699., 14 653.}	81.0155	0.799754	newly 55 weeks cumul	87 509.	14 259.9	{2413., 14 653.}	84.8849	1.00712
44	newly 3 weeks spec	15 502.	187.72	{0., 467.}	98.7891	2.7291	newly 3 weeks cumul	33 206.	6484.59	{865., 18 107.}	80.8286	0.940035	newly 5 weeks spec	12 451.	29.16	{0., 80.}	99.7658	3.61973	newly 5 weeks cumul	61 309.	6583.48	{930., 18 114.}	89.2618	1.19037	newly 61 weeks cumul	87 960.	6616.23	{961., 18 114.}	92.4781	1.34282
45	newly 3 weeks spec	16 177.	2417.37	{1., 3836.}	93.4811	2.31992	newly 3 weeks cumul	31 598.	7537.09	{954., 24 991.}	84.3946	1.09209	newly 5 weeks spec	13 010.	937.78	{0., 1598.}	92.7919	3.04539	newly 5 weeks cumul	61 499.	10 250.4	{1040., 27 829.}	86.4341	1.31418	newly 60 weeks cumul	80 329.	11 520.3	{1040., 52 447.}	85.8128	1.39366
46	newly 3 weeks spec	10 769.	2071.96	{1., 3916.}	94.6008	2.0569	newly 3 weeks cumul	20 202.	8406.12	{1041., 27 298.}	92.7375	0.946734	newly 5 weeks spec	14 108.	1044.39	{0., 927.}	92.9999	2.98021	newly 5 weeks cumul	48 516.	11 050.8	{1041., 37 203.}	87.4948	1.22816	newly 60 weeks cumul	82 843.	12 602.9	{1041., 72 926.}	85.4749	1.40953
47	newly 3 weeks spec	12 425.	931.34	{283., 1699.}	92.5043	1.2775	newly 3 weeks cumul	24 055.	22 217.5	{8389., 68 007.}	80.8086	0.392228	newly 5 weeks spec	13 477.	211.15	{3., 210.}	98.4333	2.58274	newly 5 weeks cumul	52 391.	22 791.9	{8585., 68 345.}	71.434	0.58769	newly 62 weeks cumul	79 732.	23 276.9	{8596., 68 353.}	75.0997	0.728209
48	newly 3 weeks spec	12 536.	1277.15	{267., 1928.}	89.8121	1.20436	newly 3 weeks cumul	21 158.	21 378.8	{8781., 46 103.}	71.8349	0.31259	newly 5 weeks spec	16 548.	195.84	{5., 243.}	98.8165	2.55142	newly 5 weeks cumul	54 873.	22 050.1	{8905., 46 226.}	68.0074	0.563433	newly 62 weeks cumul	90 499.	22 208.7	{8917., 46 229.}	76.9331	0.755755
49	newly 3 weeks spec	21 183.	6324.61	{1081., 14 973.}	71.5076	0.716252	newly 3 weeks cumul	34 718.	41 512.7	{10 599., 93 282.}	67.8513	0.283989	newly 5 weeks spec	14 957.	1730.56	{81., 3626.}	88.4298	1.30181	newly 5 weeks cumul	71 735.	46 456.	{12 327., 105 591.}	56.318	0.391768	newly 64 weeks cumul	87 984.	49 075.7	{13 094., 109 527.}	55.4383	0.420778
50	newly 3 weeks spec	10 269.	502.87	{0., 1123.}	95.1198	3.04421	newly 3 weeks cumul	26 490.	11 086.6	{1431., 12 998.}	72.673	0.685006	newly 5 weeks spec	9271.	154.33	{0., 451.}	98.3353	3.40178	newly 5 weeks cumul	46 170.	11 487.4	{1996., 13 462.}	80.2859	0.865409	newly 55 weeks cumul	81 778.	12 137.7	{2973., 13 863.}	85.6528	1.04568
